# Supplementary material for: Responders to Exercise Therapy in Patients with Osteoarthritis of the Hip: A Systematic Review and Meta-Analysis
Source: Int J Environ Res Public Health. 2020 Oct 10;17(20):7380. doi: 10.3390/ijerph17207380 (PMC7600967; doi:10.3390/ijerph17207380)
Supplement: Supplementary file 1 [file ijerph-17-07380-s001.pdf]

International Journal of
Environmental Research and Public Health

Systematic review

RESPONDERS TO EXERCISE THERAPY IN PATIENTS WITH OSTEOARTHRITIS OF THE HIP: A SYSTEMATIC REVIEW AND META-ANALYSIS

Carolien H. Teirlinck [1,*], Arianne P Verhagen [1,2], Elja A.E. Reijneveld [1], Jos Runhaar [1], Marienke van Middelkoop [1], Leontien M. van Ravesteyn [2], Lotte Hermsen [3], Ingrid B. de Groot [3], Sita MA Bierma-Zeinstra [1,4]

[1] Dept. General Practice, Erasmus MC University Medical Center Rotterdam, the Netherlands
[2] Discipline of Physiotherapy, Graduate School of Health, University of Technology Sydney, Australia
[3] National Health Care Institute, The Netherlands (Zorginstituut Nederland)
[4] Dept. of Orthopedics, Erasmus MC University Medical Center Rotterdam, the Netherlands

Supplementary Material S1

Search strategy

Embase.com

(osteoarthritis/exp OR (osteoarthr* OR (degenerative NEAR/2 arthritis) OR arthrosis):ab,ti) AND (Hip/exp OR 'hip osteoarthritis'/de OR 'knee osteoarthritis'/de OR knee/de OR (hip* OR knee*):ab,ti) AND (exercise/exp OR fitness/de OR 'exercise test'/de OR 'exercise tolerance'/de OR Sport/exp OR pliability/de OR 'physical activity, capacity and performance'/exp OR physiotherapy/exp OR rehabilitation/exp OR (exertion* OR exercis* OR sport* OR ((physical or motion) NEAR/5 (fitness or therap*)) OR (physical* NEAR/2 endur*) OR ((strength* or isometric* or isotonic* or isokinetic* or aerobic* or endurance or weight*) NEAR/5 (train*)) OR aquatic* OR exercis* OR physiotherap* OR manipulat* OR kinesiotherap* OR rehab* OR (skate* or skating) OR running OR jogging OR treadmill* OR swim* OR bicycl* OR (cycle* or cycling) OR walk* OR (row or rows or rowing) OR Muscle-strength* OR Balneotherap* OR hydrotherap* OR pool-therap*):ab,ti) AND ('crossover procedure'/exp OR 'double blind procedure'/exp OR 'randomized controlled trial'/exp OR 'single blind procedure'/exp OR (random* OR factorial* OR (crossover* or cross-over*) OR placebo* OR (doubl* NEXT/1 blind*) OR (singl* NEXT/1 blind*) OR assign* OR allocat* OR volunteer*):ab,ti) NOT ([animals]/lim NOT [humans]/lim)

Medline Ovid

(exp osteoarthritis/ OR (osteoarthr* OR (degenerative ADJ2 arthritis) OR arthrosis).ab,ti.) AND (Hip/ OR "osteoarthritis, hip"/ OR "osteoarthritis, knee"/ OR knee/ OR exp Knee Joint/ OR (hip* OR knee*).ab,ti.) AND (exp exertion/ OR exp Physical Fitness/ OR exp Exercise Test/ OR exp Exercise Tolerance/ OR exp Sports/ OR exp pliability/ OR exp Physical Endurance/ OR (exertion* OR exercis* OR sport* OR ((physical or motion) ADJ5 (fitness or therap*)) OR (physical* ADJ2 endur*) OR ((strength* or isometric* or isotonic* or isokinetic* or aerobic* or endurance or weight*) ADJ5 (train*)) OR aquatic* OR exercis* OR physiotherap* OR manipulat* OR kinesiotherap* OR rehab* OR (skate* or skating) OR running OR jogging OR treadmill* OR swim* OR bicycl* OR (cycle* or cycling) OR walk* OR (row or rows or rowing) OR Muscle-strength* OR Balneotherap* OR hydrotherap* OR pool-therap*).ab,ti.) AND (randomized controlled trial.pt. OR controlled clinical trial.pt. OR randomized.ab. OR placebo.ab. OR drug therapy.fs. OR randomly.ab. OR trial.ab. OR groups.ab.) NOT (exp animals/ NOT humans/)

Cochrane

((osteoarthr* OR (degenerative NEAR/2 arthritis) OR arthrosis):ab,ti) AND ((hip* OR knee*):ab,ti) AND ((exertion* OR exercis* OR sport* OR ((physical or motion) NEAR/5 (fitness or therap*)) OR (physical* NEAR/2 endur*) OR ((strength* or isometric* or isotonic* or isokinetic* or aerobic* or endurance or weight*) NEAR/5 (train*)) OR aquatic* OR exercis* OR physiotherap* OR manipulat* OR kinesiotherap* OR rehab* OR (skate* or skating) OR running OR jogging OR treadmill* OR swim* OR bicycl* OR (cycle* or cycling) OR walk* OR (row or rows or rowing) OR Muscle-strength* OR Balneotherap* OR hydrotherap* OR pool-therap*):ab,ti)

Web of science

TS=(((osteoarthr* OR (degenerative NEAR/1 arthritis) OR arthrosis)) AND ((hip* OR knee*)) AND ((exertion* OR exercis* OR sport* OR ((physical or motion) NEAR/4 (fitness or therap*)) OR (physical* NEAR/1 endur*) OR ((strength* or isometric* or isotonic* or isokinetic* or aerobic* or endurance or weight*) NEAR/4 (train*)) OR aquatic* OR exercis* OR physiotherap* OR manipulat* OR kinesiotherap* OR rehab* OR (skate* or skating) OR running OR jogging OR treadmill* OR swim* OR bicycl* OR (cycle* or cycling) OR walk* OR (row or rows or rowing) OR Muscle-strength* OR Balneotherap* OR hydrotherap* OR pool-therap*)) AND ((random* OR factorial* OR (crossover* or cross-over*) OR placebo* OR (doubl* NEAR/1 blind*) OR (singl* NEAR/1 blind*) OR assign* OR allocat* OR volunteer*)))

CINAHL EBSCOhost

(MH osteoarthritis+ OR TI (osteoarthr* OR (degenerative N1 arthritis) OR arthrosis) OR AB (osteoarthr* OR (degenerative N1 arthritis) OR arthrosis)) AND (MH Hip OR MH "osteoarthritis, hip" OR MH "osteoarthritis, knee" OR MH knee OR MH "Knee Joint" OR (hip* OR knee*)) AND (MH exertion+ OR MH Physical Fitness+ OR MH Exercise Test+ OR MH Exercise Tolerance+ OR MH Sports+ OR MH pliability+ OR MH Physical Endurance+ OR TI (exertion* OR exercis* OR sport* OR ((physical or motion) N4 (fitness or therap*)) OR (physical* N1 endur*) OR ((strength* or isometric* or isotonic* or isokinetic* or aerobic* or endurance or weight*) N4 (train*)) OR aquatic* OR exercis* OR physiotherap* OR manipulat* OR kinesiotherap* OR rehab* OR (skate* or skating) OR running OR jogging OR treadmill* OR swim* OR bicycl* OR (cycle* or cycling) OR walk* OR (row or rows or rowing) OR Muscle-strength* OR Balneotherap* OR hydrotherap* OR pool-therap*) OR AB (exertion* OR exercis* OR sport* OR ((physical or motion) N4 (fitness or therap*)) OR (physical* N1 endur*) OR ((strength* or isometric* or isotonic* or isokinetic* or aerobic* or endurance or weight*) N4 (train*)) OR aquatic* OR exercis* OR physiotherap* OR manipulat* OR kinesiotherap* OR rehab* OR (skate* or skating) OR running OR jogging OR treadmill* OR swim* OR bicycl* OR (cycle* or cycling) OR walk* OR (row or rows or rowing) OR Muscle-strength* OR Balneotherap* OR hydrotherap* OR pool-therap*)) AND (PT randomized controlled trial OR PT controlled clinical trial OR AB (randomized OR placebo OR randomly OR trial OR groups)) NOT (MH animals NOT MH humans)

Sensitivity analysis

	Experimental		Control			Risk Difference
Study or Subgroup	Events	Total	Events	Total	Weight	M-H, Random, 95% CI
**After treatment, short term**						
Bieler 2016	19	48	5	43	6.2%	0.28 [0.11, 0.45]
Fernandes 2010	15	55	7	54	7.0%	0.14 [-0.00, 0.29]
French 2013	17	44	13	43	5.3%	0.08 [-0.11, 0.28]
Hermann 2016	14	38	4	39	5.8%	0.27 [0.09, 0.45]
Hopman-Rock 2000	3	14	5	18	3.1%	-0.06 [-0.36, 0.23]
Juhakoski 2011	9	60	10	58	7.6%	-0.02 [-0.16, 0.11]
Krauss 2014	21	65	11	67	7.1%	0.16 [0.01, 0.30]
Saw 2016	9	13	2	11	2.6%	0.51 [0.17, 0.85]
Tak 2005	3	45	8	44	7.5%	-0.12 [-0.25, 0.02]
Teirlinck 2016	30	88	12	85	7.9%	0.20 [0.08, 0.32]
v Baar 1998	28	40	18	38	4.9%	0.23 [0.01, 0.44]
Villadsen 2014	9	41	1	34	7.3%	0.19 [0.05, 0.33]
**Subtotal (95% CI)**		**551**		**534**	**72.3%**	**0.14 [0.06, 0.23]**
Total events	177		96			
Heterogeneity: Tau² = 0.01; Chi² = 32.77, df = 11 (P = 0.0006); I² = 66%						
Test for overall effect: Z = 3.30 (P = 0.0010)						
**6-8 months after treatment, long term**						
Abbott 2013	9	22	5	23	3.7%	0.19 [-0.07, 0.46]
Bieler 2016	12	40	7	34	5.3%	0.09 [-0.10, 0.29]
Fernandes 2010	11	48	5	42	6.7%	0.11 [-0.04, 0.26]
Saw 2016	5	11	3	11	2.0%	0.18 [-0.21, 0.58]
Stener-Victorin 2004	4	9	0	5	2.2%	0.44 [0.06, 0.82]
Teirlinck 2016	27	87	14	79	7.7%	0.13 [0.00, 0.26]
**Subtotal (95% CI)**		**217**		**194**	**27.7%**	**0.14 [0.06, 0.22]**
Total events	68		34			
Heterogeneity: Tau² = 0.00; Chi² = 3.03, df = 5 (P = 0.70); I² = 0%						
Test for overall effect: Z = 3.47 (P = 0.0005)						

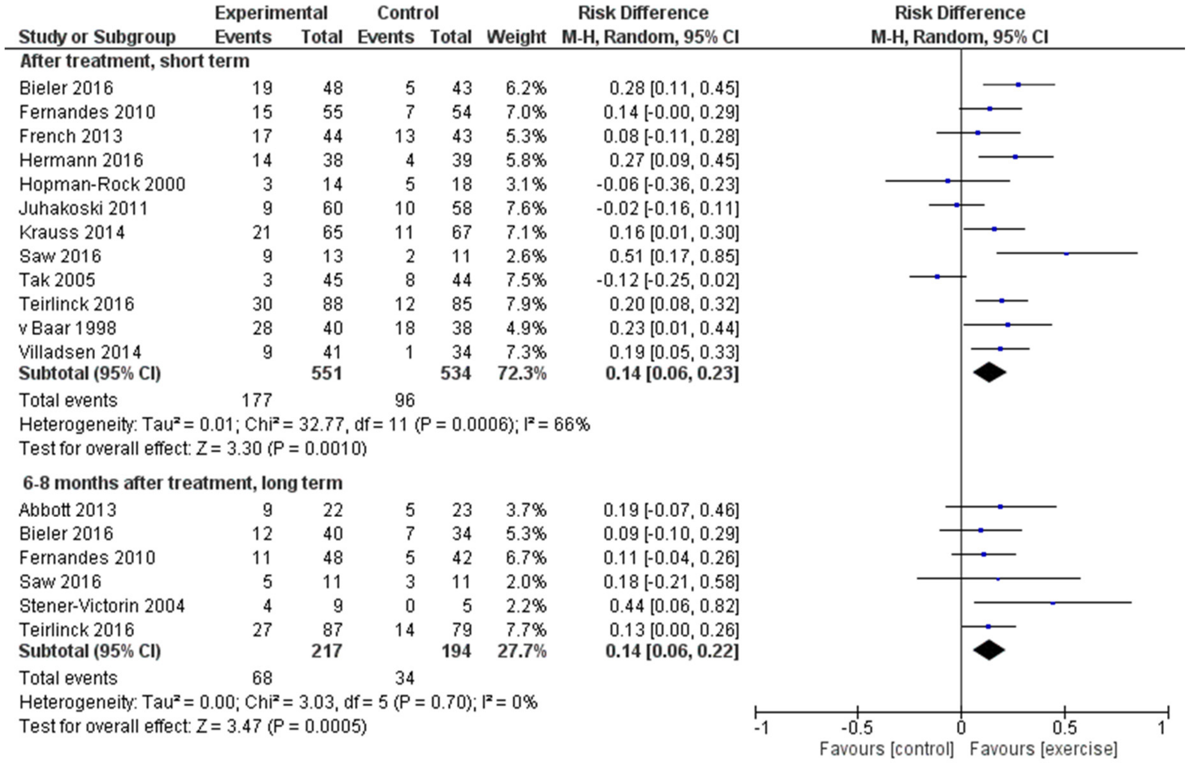

Figure S1 Forest plot meta-analysis, complete cases (no missing data)

	Experimental		Control			Risk Difference
Study or Subgroup	Events	Total	Events	Total	Weight	M-H, Random, 95% CI
**After treatment, short term**						
Bieler 2016	19	50	5	52	12.9%	0.28 [0.13, 0.44]
French 2013	17	45	13	43	8.1%	0.08 [-0.12, 0.27]
Teirlinck 2016	30	101	12	102	26.7%	0.18 [0.07, 0.29]
v Baar 1998	28	41	18	40	7.2%	0.23 [0.02, 0.44]
**Subtotal (95% CI)**		**237**		**237**	**54.9%**	**0.20 [0.12, 0.27]**
Total events	94		48			
Heterogeneity: Tau² = 0.00; Chi² = 2.86, df = 3 (P = 0.41); I² = 0%						
Test for overall effect: Z = 5.05 (P < 0.00001)						
**6-8 months after treatment, long term**						
Abbott 2013	9	22	5	23	4.5%	0.19 [-0.07, 0.46]
Bieler 2016	12	50	7	52	14.0%	0.11 [-0.05, 0.26]
Teirlinck 2016	27	101	14	102	26.6%	0.13 [0.02, 0.24]
**Subtotal (95% CI)**		**173**		**177**	**45.1%**	**0.13 [0.04, 0.21]**
Total events	48		26			
Heterogeneity: Tau² = 0.00; Chi² = 0.31, df = 2 (P = 0.86); I² = 0%						
Test for overall effect: Z = 3.01 (P = 0.003)						

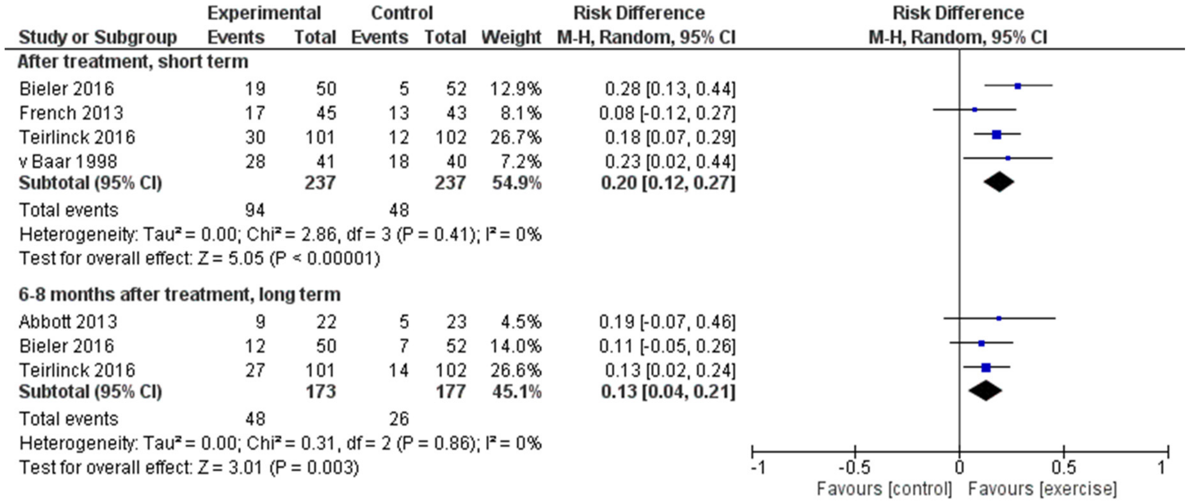

Figure S2. Forest plot, meta-analysis with only studies with global assessment
